# Pollution, Particles, and Dementia: A Hypothetical Causative Pathway

**DOI:** 10.3390/ijerph17030862

**Published:** 2020-01-30

**Authors:** Anthony Seaton, Lang Tran, Ruoling Chen, Robert L. Maynard, Lawrence J. Whalley

**Affiliations:** 1 Department of Environmental Medicine, Aberdeen University, Aberdeen AB25 2ZP, UK; 2 Institute of Occupational Medicine, Edinburgh EH14 4AP, UK; 3 Department of Public Health, Wolverhampton University, Wolverhampton WV1 1LY, UK; R.Chen@wlv.ac.uk; 4 Department of Environmental Science, Birmingham University, Birmingham B15 2TT, UK; 5 Department of Mental Health, Aberdeen University, Aberdeen EH14 4AP, UK

**Keywords:** particulate air pollution, dementia, endothelial inflammation, biologic microparticles, blood-brain barrier

## Abstract

Epidemiological studies of air pollution have shown associations between exposure to particles and dementia. The mechanism of this is unclear. As these seem unlikely in terms of the very small dose likely to reach the brain in usual Western urban circumstances, we extend our 1995 hypothetical explanation of the association of air pollution with cardiac deaths as a plausible alternative explanation of its associations with dementia. Since our original proposal, it has become apparent that inflammation may be carried by blood from organ to organ by biologic microparticles derived from cell membranes. These transmit inflammatory messages to endothelial cells throughout the body as part of a general defensive response to assumed bacterial infection; particulate air pollution has recently been shown to be associated with their release into the blood. We propose that episodic release of biologic microparticles from pollution-induced lung inflammation causes secondary inflammation in the blood-brain barrier and cerebral microbleeds, culminating over time in cognitive impairment. Ultimately, by incomplete repair and accumulation of amyloid, this increases the risk of Alzheimer’s disease. Importantly, this mechanism may also explain the relationships of other inflammatory conditions and environmental factors with cognitive decline, and point to new opportunities to understand and prevent dementia.

## 1. Introduction

High air pollution episodes associated with fossil fuel combustion have been linked to excess deaths in the exposed population since the 1930s [1]. In the 1990s, such excesses were reported at much lower urban pollution concentrations, not confined to smog episodes [2,3]. The excesses were from lung and heart disease and the risk was related mainly to particle concentrations; lung deaths were explicable in terms of lung inflammation, but the more frequent cardiac deaths less easily. Exposures of the *lung* to largely carbon particles, at doses of only 1 or 2 mg over 24 hours, were associated with death or hospitalization from *heart attack*. More recently, similar relationships have been shown with dementia [4,5,6]. In both cases a dose-related causative association does not seem plausible toxicologically, yet the epidemiologic evidence seems consistent, notably among the elderly [7].

### The Original Hypothesis

In 1995 we published a hypothetical explanation of the cardiac deaths; the lung takes account not of the mass of foreign material to which it is exposed, but rather of the numbers of particles; that is, it does not discriminate between bacterial-sized particles but treats them similarly—the greater numbers deposited, the greater the apparent threat and the stronger the response [8]. Behind this was the understanding that most pollution particles are of very small size, in the nanometer range (below 0.1 µm diameter) shown by toxicologists to have different effects on the lung than the same material of larger size when inhaled [9]. It was also known that population fibrinogen concentrations in the blood (a marker of inflammation) rise in winter when pollution is higher. The hypothesis proposed that the lung responds by inflammation mediated by macrophages and cytokine release, which then has downstream effects on blood coagulability and thus risk of myocardial infarct. In 1999, following our observation of falls in red blood cell and platelet counts in association with rises in particulate air pollution in healthy elderly people, we extended this hypothesis to propose that the vascular endothelial cell was central to the response to pollution [10]. These early observations had a significant impact, notably on risk assessment of nanotechnologies [11,12]. 

## 2. Discussion

### 2.1. Explaining the Epidemiologic Associations

Much subsequent epidemiologic and toxicologic investigation has supported the concept that very small particles, usually measured as the mass (in micrograms of those less than 2.5 µm in aerodynamic diameter, PM_2.5_), inhaled in very large numbers but low mass can increase risks of myocardial infarct, stroke, and venous thrombosis [13]. Lung inflammation, involving free radical release from particle surfaces, depends on particle type and surface chemistry [14], but the mechanisms of transfer of inflammatory messages to other organs remain unclear; it is unlikely that they operate solely through the clotting system. However, the emphasis of the original hypothesis has been changed as research has concentrated more on the special properties of nanoparticles than on mechanisms of transfer of inflammatory messages; this has influenced public understanding. The media and the public have now accepted an extension of the hypothesis that may be expressed thus: *very small particles after inhalation are able to move via the blood to every organ in the body, where they may be assumed to set up inflammation and cause cellular damage* [15]. 

Epidemiologists are now finding associations between air pollution exposure in populations and possible harm to the brain or even the fetus [4,7,16]. Experimentally, inhaled nanoparticles may be found in the blood and even in the brain, derived either from pulmonary or olfactory nerve deposition [17,18]. However, assuming a daily ventilation rate of 11 m^3^, a lifetime of breathing air containing 20 micrograms per cubic meter of small particles (PM_2.5_), double the World Health Organization annual average guideline, will involve the inhalation of a total of about 6 grams of dust, about a teaspoonful, *over 80 years*. Half of this is immediately exhaled and of that retained less than 1% is transferred to the blood stream [19,20], where it is largely cleared by phagocytosis. No chemical poison administered over a lifetime could cause injury in such tiny doses. This must lead to some skepticism of the multiple frightening consequences of breathing polluted air in places of relatively low pollution, such as most of the USA. An amplification signal is required to explain this association, even when it is realized that a low average concentration over years will always include episodes of much higher concentrations capable of causing transient lung inflammation.

### 2.2. From Lung to Other Organs; How Is the Message Sent?

The original hypothesis relied on an amplifying effect of lung inflammation caused by huge numbers of nanoparticles, misinterpreted by the lung as bacteria with their potential to invade and multiply, to explain the toxicity of a low mass of apparently non-toxic matter. Increased coagulability was the mechanism proposed but later, based on the serendipitous finding of transient sequestration of red blood cells and platelets in individuals as airborne particle concentrations rose, we suggested that particle exposure also caused activation of endothelial cell functions [10]. The implicit extension to the original hypothesis, that *small numbers* of tiny combustion particles transferred to the distal organs can have such effects seems implausible on the grounds of dosage and we propose an alternative to explain the observed associations as part of a wider biologic explanation of the effects of inflammation. As such, it is testable epidemiologically and experimentally and may open fruitful new directions for research. 

### 2.3. Biologic Microparticles

Recently, the concept of local inflammation producing small inflammogenic particles derived from activation or apoptosis of endothelial and other cells (confusingly called microparticles) has been developed [21,22,23]. These *biological* particles are over 100 µm in diameter, are fragments of cell membrane, and transfer both pro- and anti-inflammatory messages through the blood stream to endothelial cells. They provide a plausible explanation of downstream effects; indeed, they have already been shown to be released in populations in relation to high episodic rises in air pollution as a contributory factor in vascular disease [24]. Inflammation has both local and general effects designed to overcome invasion by microorganisms. The release of these sub-cellular particles that transmit inflammatory messages via the blood to other organs is part of this reaction. It is a teleologically understandable means of passing warning signals through the blood stream to other organs but, as with all defensive inflammatory events, may have negative effects [25]. In extreme cases, such as sepsis, these may be fatal, but smaller chronic effects could promote atheroma or damage to the small vessels of the brain and the blood-brain barrier. 

### 2.4. Environmental Risk Factors for Dementia

There is increasing interest in environmental risk factors for dementia. Many are common to those for cardiac disease, namely socioeconomic deprivation, low level of education, tobacco and alcohol consumption, poor diet (leading to obesity), and lack of exercise [26]. There are also medical risk factors, notably head injury, chronic obstructive pulmonary disease (COPD), hypertension, angina, delirium, diabetes, and acute inflammatory episodes [27,28]. Furthermore, there is evidence that the use of statins protects against both heart disease and dementia [29]. This suggests that lifestyle factors, potentially susceptible to public health measures, may be influenced in order to decrease the future incidence of dementia. However, if such diverse factors all increase risks of damage to brain cells, what is the final common pathway? 

There is convincing evidence of an association between rises in particulate air pollution and risk of stroke [30]. Combustion-generated air pollution particles, in sufficient dose, are a cause of lung and systemic inflammation and the secondary effects of this on blood coagulability and the brain vasculature could precipitate stroke *as an acute effect*, by analogy with their association with heart disease. However, evidence of associations of pollution with dementia and cognitive decline is strongly suggestive of a mechanism adding to risk *over the life course* [5,7,16]. While much research on dementia has concentrated on factors relating to the effects and causation of structures seen on histopathology of the damaged brain, another line has investigated the relevance of the small blood vessels of the brain. Recognition of environmental influences in dementia has led to a call for integration of the epidemiologic and toxicologic research [31]. Inflammation is a key component of conditions such as hypertension, obesity, and diabetes and is associated with the release of biological microparticles into the blood, which may transmit inflammation from one location to another and contribute to the persistence of inflammation locally. Raised blood concentrations of biological microparticles have been found in individuals with Alzheimer’s disease [32,33], and their deposition on cerebral vascular endothelium would be expected to lead to inflammation and microbleeds. Such brain microbleeds, forerunners of dementia, have been demonstrated analogously in chronic obstructive pulmonary disease, a condition also associated with generation of microparticles [34,35]. 

## 3. Conclusions

### The Hypothesis

We propose that chronic exposure of the small blood vessels of the brain to biological microparticles leads to local inflammation and microhemorrhages at the blood brain barrier. In the absence of collagen-producing cells at the cerebral capillary level in the brain, repair is dependent upon neurons and astrocytes producing an alternative cell adhesion protein, amyloid, for scaffold re-construction. This is a short chain of about 40 amino acids and is neurotoxic, cleaved from amyloid precursor protein, a transmembrane protein with roles in neurogenesis as both a cell surface recognition and adhesion molecule. Amyloid appears to be proinflammatory either by activating microglia or directly on neurovascular endothelium [36]. Over-production and polymerization are the basis of the amyloid plaque. The figure demonstrates that amyloid can either repair the damage to the blood-brain barrier or initiate further inflammation and amyloid production. Analogous to atheroma formation in large vessel disease, these plaques can be either stable or unstable. For unstable plaques, there is no inflammatory resolution and continuing microglial release of pro-inflammatory cytokines drives the neurons eventually to neurodegeneration. Once part of the blood-brain barrier becomes inflamed, it is likely that more microparticles are produced and released locally by endothelial cells and platelets, and the process becomes self-replicating. Thus, a vicious circle of inflammation, capillary damage and further inflammation silently compromises cerebral function. If the normal removal of amyloid by the blood is impaired by the same vascular inflammatory process, accumulation leads to death of neurons and ultimately the clinico-pathological features of Alzheimer’s disease. The site of maximal deposition of microparticles would be dependent on local blood flow, influenced both by anatomical and physiological factors, particularly neuronal activity.

This hypothesis explains the observed associations between air pollution and cognitive decline, based on recurrent episodes over years of apparently minor lung inflammation caused by particulate pollution. Substantial rises in pollution particle mass concentrations have been shown to be associated with increased blood levels of microparticles derived from endothelial cells [24]. Similar hypotheses could explain associations between environmental factors and disease of other organs or effects on the developing fetus. Since these biological microparticles may also be released in excess in COPD, hypertension, sepsis, diabetes, and obesity, all conditions associated with increased risk of dementia, the hypothesis may have broader application in research into prevention and amelioration of dementia by taking account of the role of inflammation and its environmental causes.

This hypothesis may be tested in experimental models and is likely to be incorporated in future epidemiologic studies of air pollution and its widespread associations. 
